# A multiscale stochastic cellular automata model for dispersion of mountain pine beetles

**DOI:** 10.1098/rsos.241934

**Published:** 2025-06-18

**Authors:** Yanjun Liu, Donald Estep

**Affiliations:** ^1^ Statistics and Actuarial Science, Simon Fraser University, Burnaby, British Columbia, Canada

**Keywords:** dispersion process, multiscale modelling, stochastic cellular automata, calibration, mountain pine beetle

## Abstract

We construct a multiscale model of dispersion of mountain pine beetles in western North American forests. Dispersion is modelled at a macroscale (regional scale) by a novel nonlinear stochastic process for population density. This is coupled to a microscale (tree scale) model of population dynamics using a sequential approach, which assumes that dispersion occurs on a slower time scale than local dynamics. We conduct a numerical investigation of the multiscale model properties. While the model depends on a relatively small number of parameters, it can produce a wide range of behaviours, including dispersion patterns qualitatively similar to those of mountain pine beetle infestations. We also conduct a Bayesian calibration for key parameters in the model using synthetic and real data.

## 1. Introduction

**Figure 1.**
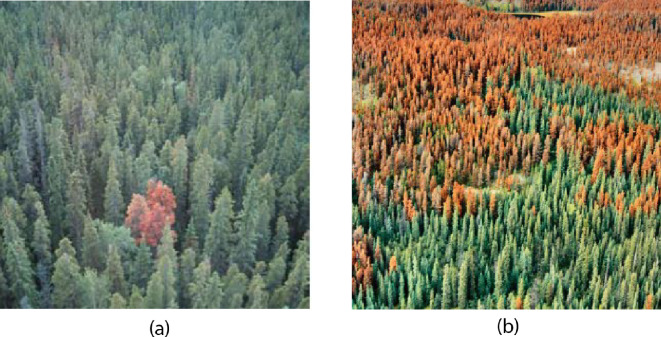
Aerial view of trees infested by MPBs. (a) A small group of tree infestations in the endemic stage. Photo is taken from [10]. (b) Large patches of infestation in the epidemic stage. Photo taken from [5].

The mountain pine beetle (MPB*, Dendroctonus ponderosae*) is an integral part of the forest ecosystem throughout western North America [1–9]. In dormant periods, MPB infestations affect sparse collections of weakened trees and cause little damage. But cyclically, MPB populations reach epidemic levels during which MPB attacks damage healthy trees over extensive areas (figure 1). These epidemics have significant ecological and economic consequences, including widespread tree mortality, wildfire threats, forest structure and composition change, and threats to watersheds and wildlife [10–16].

The MPB is univoltine with a life cycle that is correlated annually with the temperature, though MPB may re-emerge to develop a second brood if the temperature is sufficiently elevated [1]. MPB has the following four life stages: egg, larva, pupa and adult. The first three stages take place within the subcortical tissues of host trees [3]. However, during the warm summer months, young adult beetles leave their current host tree to disperse to potential new hosts. Trees defend themselves by releasing toxic resins, and mass attack is crucial to successful infection as it enables beetles to overcome the tree defences. The defences are weakened when the trees are under stress such as drought, which is one reason weather and climate play a key role in the MPB population cycle.

There are four major phases in the MPB population cycle [5,17]. In endemic phases, the MPB populations are small, and they are limited to a few weakened trees. As the populations grow to a size that can overcome defences of healthy trees, the population enters an incipient-epidemic phase. Epidemics occur when there are large, contiguous areas of susceptible host trees and favourable weather conditions. The post-epidemic phase is characterized by the decline in MPB populations.

In the endemic phase, dispersion is slow and infestation is limited to isolated trees (figure 1). As the population grows, infestation affects collections of trees, and the population enters the epidemic phase. The epidemic stage can last several years with favourable weather conditions and a sufficient number of susceptible host trees [18–22].

While MPB dispersion generally occurs over short ranges, beetles may travel further under natural conditions and human-facilitated transport [3,5,23]. The patterns of affected and unaffected trees in a region are generally heterogeneous in all but very late stages of an epidemic (figure 1). This reflects the complex factors affecting conditions for dispersion.

In general, *dispersion*, or the collective spatial movement of individuals in a population, often plays a prominent role in population dynamics [3,24–29]. Dispersion is generally a multiscale phenomenon bridging microscale processes affecting individuals with macroscale processes affecting collections of individuals. For example, dispersion of MPB is affected by the population dynamics of individual MPB that takes place on the tree scale as well as macroscale factors such as forest cover, wind and terrain.

Modelling of multiscale phenomena presents well-known challenges in many disciplines [30]. For example, even when there is a good understanding of behaviour at individual scales, the interactions between processes occurring at different scales are often unclear; the simulation of models of multiscale phenomena is often burdensome; and calibration of models of multiscale phenomena—determining parameters in the models from observed data—is an open research question in many cases. As a consequence, one common approach to model multiscale phenomena involves ‘upscaling’ microscale behaviour, e.g. by some form of averaging, to obtain parameters and data for a macroscale model, which is then simulated without reference to microscale dynamics. While the resulting macroscale model may describe bulk properties of the multiscale phenomena with great computational efficiency, such an approach can fail to quantify the impact of dynamics at the microscale level on system behaviour. For this reason, ‘multiscale’ models that couple microscale and multiscale models are appealing. However, any such model must address the aforementioned challenges.

In this paper, we construct and investigate a multiscale stochastic model for dispersion that couples a stochastic microscale population dynamics model with a stochastic macroscale model for dispersion [29,31]. While the dispersion model is general in construction, it is motivated by MPB dispersion. The macroscale stochastic model for dispersion conforms to the observation that the likelihood of dispersion into a location increases when the MPB density is elevated in nearby locations but dispersion does not progress in a deterministic uniform fashion. At the individual tree scale (the *microscale*), we use an ecology-informed model that describes population dynamics of the insects.

There is a rich and varied literature on models for MPB population dynamics and dispersion (e.g. [23,24,27,32–42]). The models span the range from statistical descriptions of MPB mortality such as life tables informed by observational data to process models, e.g. differential equations, describing population dynamics and dispersion. Existing models focus on behaviour at a single scale. Models at the tree scale tend to be informed by detailed biological knowledge of the MPB, and trees and the models can often be validated against experimental observation. Previous models of MPB dispersion and aggregation tend to be based on general assumptions including the following: (i) pioneer beetles attack randomly over the available trees; (ii) there is a threshold of beetle population needed to induce aggregation on a tree; (iii) any tree affected by beetle aggregation is killed. For example, Burnell [24] developed a stochastic model for MPB infestation that assumes that pioneer MPB attacks a tree randomly over its available surface, and there is a threshold of aggregation of pioneers above which there is sufficient population to induce mass aggregation, which eventually kills the tree.

Novel features of our multiscale model include the following: (i) a new stochastic macroscale model for the dispersion of the population density across large regions; (ii) the modelling framework can incorporate any microscale model that describes individual behaviour in very small areas; (iii) the microscale and macroscale models are coupled using a sequential approach that allows for wide differences in spatial and temporal scales between microscale and macroscale; and (iv) the model depends on a relatively small number of parameters that have physical interpretations and can be calibrated from observational data, yet the model can exhibit a wide range of behaviours.

In §2, we construct the multiscale stochastic model and describe the application to MPB. In §3, we conduct a numerical investigation of the behaviour and properties of the multiscale dispersion model. In §4, we discuss calibration of parameters in the macroscale stochastic model using synthetic and real data. We present a conclusion in §5.

## 2. The multiscale model for dispersion

At the macroscale, we model dispersion by a *stochastic cellular automata* (SCA), which is a nonlinear discrete-time stochastic process that uses probabilistic rules to determine the flow of population density between members of a collection of cells that partitions the region under consideration. The SCA is inspired by the ‘Game of Life’ [43], which is a cellular automata [29,31,43–52] that evolves a system that takes values of 0 and 1 on a grid of square cells. The values can be interpreted as indicating ‘not populated’ or ‘populated’, respectively. In a sequence of time steps, the rules evolving the value in a given cell depend on the values of its eight neighbouring cells, e.g. if there are too many neighbours with value 1, the value of the cell is set to 0 because the neighbourhood is ‘over-populated’. The Game of Life is deterministic with the subsequent behaviour determined by the initial pattern. Nonetheless, it produces interesting patterns and dynamical behaviour.

The application to dispersion of a population like MPB suggests some alterations to the Game of Life. Generally, the model should allow for a more refined description of population than ‘populated or not’, and it should allow for randomness in the dynamics. Specifically, the model should have the following features:

— The state in each cell should be defined as a density between the values of 0 representing no population and 1 representing population at a maximum capacity.
— The changes to states should be expressed as fractional changes to density.
— While the rules determining the probability of dispersion should be deterministic, the process governing changes to cell states should be stochastic.
— The model should allow for a source of population independent of the dynamics of the dispersion and the initial state.

Our model incorporates these features. Each cell in the partition defining the SCA contains the state representing the (average) density of a population in the cell. The SCA defines a procedure that determines the state in each cell at a given time step based on the states of the cell and some of its neighbours at the previous time step. The procedure uses a stochastic process to determine cell states using deterministic rules that depend on parameters that can be interpreted as reflecting mortality, reproduction, effect of distance and the chance of successful colonization. All cells use the same update rule, but parameters may vary across cells and time. The macroscale dispersion model couples to a microscale population dynamics model that models the production of population.

### 2.1. The macroscale stochastic cellular automata model for dispersion

#### 2.1.1. Partitioning the domain

**Figure 2.**
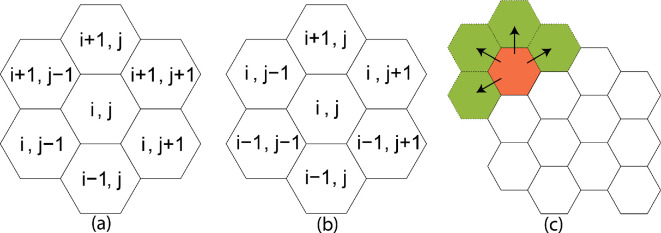
(a) The indexing for adjacent cells when the column index j is odd. (b) The indexing for adjacent cells when the column index j is even. (c) An example of auxiliary cells (green) adjacent to a boundary cell (orange).

We use a partition of a fixed spatial domain comprised of hexagonal cells. We let $Z^{2}=\{\kappa_{ij}{\}}_{i=1,j=1}^{m,n}$ denote the disjoint collection of m×n uniformly sized cells organized in m rows and n columns. The benefits of using hexagonal cells include relatively efficient high-fidelity descriptions of geographic features that can be constructed, and the distance between centroids of hexagonal cells is the same in six directions, thus reducing directional bias in dispersion. (The impact on dynamics of using a rectangular partition of the domain for the Game of Life is visibly evident in simulations.) A disadvantage is that indexing the cells is more complicated than for a rectangular grid (figure 2). Cell states at time t are represented by normalized population densities $\{\pi_{ij}^{\left(t\right)}{\}}_{(i,j)\in Z^{2}}$ taking values in the interval Π=[0,1]*,* so the configuration space is $Q=\Pi^{Z^{2}}$.

#### 2.1.2. Determining the interaction neighbourhood

**Figure 3.**
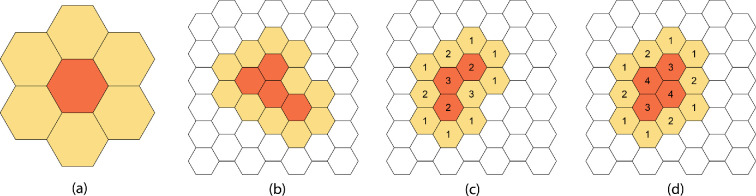
(a) The Moore neighbourhood of the central dark-shaded cell. (b) An interaction neighbourhood showing dark-shaded affected cells and their unaffected neighbours. (c,d) The inclusion weights $w_{ij}^{\left(t\right)}$ for cells in an interaction neighbourhood at subsequent time steps. The number in each cell represents its inclusion weight. (c) An interaction neighbourhood with three affected cells. (d) The updated interaction neighbourhood at the next time step when one more cell becomes affected.

Dispersion occurs between cells within an ‘interaction neighbourhood’. The building block is the *Moore neighbourhood* of a cell, which is the cell and its six neighbouring cells (figure 3). The density in a cell only potentially disperses to cells in its Moore neighbourhood. We group all cells that potentially interact through dispersion at a given time step in an *interaction neighbourhood*, defined as a group of *affected* (non-zero density) cells that are not separated by more than two non-affected (zero density) cells along with all immediate neighbours (figure 3). Dispersion in distinct interaction neighbourhoods occurs independently.

#### 2.1.3. The framework for dispersion

The dispersion of density between cells is governed by a stochastic process that determines how the density changes over a time step according to a fixed set of rules. The SCA evolves the cell densities on $Z^{2}$ over *discrete times* t=0,1,2,⋯ corresponding to a *macroscale time step* Δt=1, where we assume Δt=1 without loss of generality. The change in density $\pi_{ij}^{\left(t\right)}$ of cell $\kappa_{ij}$ is computed on the basis of several random draws from probabilities that depend on the densities of the cells in the interaction neighbourhood of $\kappa_{ij}$. Since dispersion in a given interaction neighbourhood occurs independently of dispersion in other interaction neighbourhoods, we can describe dispersion within a given interaction neighbourhood without loss of generality.

#### 2.1.4. The probability of inclusion in dispersion

Dispersion is determined by outcomes of random sampling from a series of probabilities. The first in the series is the *inclusion probability*, denoted by $p_{I,ij}$*,* which is the probability that cell $\kappa_{ij}$ is included in the dispersion process, either by contributing or receiving dispersed population, at this time. There are two considerations. First, we assume that the probability of a cell being included in dispersion increases as number of neighbouring affected cells increases. Second, we assume that larger relative density difference in the cells in an interaction neighbourhood yields a higher probability of dispersion occurring. We define


(2.1) $$\begin{array}{lcr} & p_{I,ij}^{\left(t\right)}=\frac{w_{ij}^{\left(t\right)}}{1+w_{ij}^{\left(t\right)}}\times d_{ij}^{\left(t\right)}, & \end{array}$$

where $d_{ij}^{\left(t\right)}$ is equal to the maximum value of the density difference between $\kappa_{ij}$ and its six neighbouring cells at time t, and the *inclusion weight* $w_{ij}^{\left(t\right)}$ determines the likelihood of inclusion in dispersion for $\kappa_{ij}$ at time t. For non-affected cells, the inclusion weight $w_{ij}^{\left(t\right)}$ is calculated as the number of adjacent affected neighbours at each time step. Affected cells are more likely to be involved in dispersion process, so the inclusion weight $w_{ij}^{\left(t\right)}$ for affected cells is the number of its adjacent affected neighbours plus 1 (figure 3). For an isolated affected cell, the probability is half of the density in the cell. As the inclusion weight increases, the probability increases towards the maximum difference in density.

Inclusion in dispersion at time t is determined by random draw. For an affected cell $\kappa_{ij}$ at time t, we define the Bernoulli random variable $B_{I,ij}^{\left(t\right)}$ that indicates if the cell is included in dispersion ($B_{I,ij}^{\left(t\right)}=1$) or not, where


$$B_{I,ij}^{\left(t\right)}=\left\{ \begin{array}{ll} 1, & \text{with probability }p_{I,ij}^{\left(t\right)}\text{,}\\ 0, & \text{with probability }1-p_{I,ij}^{\left(t\right)}\text{.} \end{array}\right.$$

During a simulation, we draw a sample $B_{I,ij}^{\left(t\right)}$, and only cells with $B_{I,ij}^{\left(t\right)}=1$ are included in dispersion.

In a more general form of the model [29], the parameters may vary across cells and time steps to reflect spatial and temporal inhomogeneity of physical conditions. In addition, we can introduce parameters $\alpha_{ij}^{\left(t\right)}$ and $\beta_{ij}^{\left(t\right)}$ to emphasize or de-emphasize the effect of differences in weights and density. The general form of the model is


(2.2) $$\begin{array}{lcr} & p_{I,ij}^{\left(t\right)}={\left[\frac{{\left(w_{ij}^{\left(t\right)}\right)}^{\alpha_{ij}^{\left(t\right)}}}{1+{\left(w_{ij}^{\left(t\right)}\right)}^{\alpha_{ij}^{\left(t\right)}}}d_{ij}^{\left(t\right)}\right]}^{\beta_{ij}^{\left(t\right)}}, & \end{array}$$

with $\alpha_{ij}^{\left(t\right)}>0$ and $\beta {ij}^{\left(t\right)}>0$.

#### 2.1.5. Determining the amount of potential dispersion

At time t, we divide the cells in a given interaction neighbourhood that are included in the dispersion into *contributing cells*, which are the cells whose density is higher than the *mean density* ${\bar{\pi }}^{\left(t\right)}$ of the included cells in the interaction neighbourhood, and the *non-contributing cells*, which are the rest. We assume that all of the dispersed population comes from contributing cells and the dispersed population disperses uniformly to all of the cells in the interaction neighbourhood included in the dispersion process. (In a typical application to MPB dispersion, a cell contains multiple trees, so some MPB disperse to other trees in the same cell and others disperse to trees in the neighbouring cells.) The contributing cells are denoted $\{\kappa_{i_{h}\;j_{h}}^{\left(t\right)}{\}}_{h=1}^{H^{\left(t\right)}}$, where $H^{\left(t\right)}$ is the number of contributing cells and $\{i_{h}\;j_{h}{\}}_{h=1}^{H^{\left(t\right)}}$ is a subset of distinct indices of cells included in dispersion. A contributing cell satisfies $\pi_{i_{h}\;j_{h}}^{\left(t\right)}\geq {\bar{\pi }}^{\left(t\right)}$. The non-contributing cells are denoted $\{\kappa_{{\hat{i}}_{k}\;{\hat{j}}_{k}}^{\left(t\right)}{\}}_{k=1}^{K^{\left(t\right)}}$, where $K^{\left(t\right)}$ represents the number of non-contributing cells at time t and $\{{\hat{i}}_{k}\;{\hat{j}}_{k}{\}}_{k=1}^{K^{\left(t\right)}}$ is a subset of distinct indices of cells included in dispersion that does not intersect $\{i_{h}\;j_{h}{\}}_{h=1}^{H^{\left(t\right)}}$. Here, $H^{\left(t\right)}+K^{\left(t\right)}$ equals the number of cells in the interaction neighbourhood of $\kappa_{ij}$ that are included in the dispersion process at that time.

We allow the contributing cells to contribute a proportion of the density above ${\bar{\pi }}^{\left(t\right)}$. The total amount of density to be dispersed within the interaction neighbourhood is


$$\begin{array}{c} {\tilde{\pi }}^{\left(t\right)}=\sum_{h=1}^{H^{\left(t\right)}}\gamma_{1}(\pi_{i_{h}\;j_{h}}^{\left(t\right)}-{\bar{\pi }}^{\left(t\right)}), \end{array}$$

where the parameter $\gamma_{1}$ determines the fraction of population density to be dispersed. Cells that have a higher density contribute more.

#### 2.1.6. Computing dispersion

We consider that some of the dispersed density may be ‘lost’ during the process in two ways. Namely, some portion of the density may be lost during dispersion and some of the density that reaches a new host might not infect the host successfully. This is motivated by the field experiments conducted to investigate the dispersion of MPB described in [3] and the modelling assumptions in [24].

We assume the *dispersion survival rate* $\gamma_{2}$ is the fraction of the dispersed population that successfully reaches a new host, so $1-\gamma_{2}$ determines the population loss during dispersion. The state for contributing cells is given by the formula


$$\begin{array}{c} \pi_{i_{h}\;j_{h}}^{\left(t+1\right)}=\pi_{i_{h}\;j_{h}}^{\left(t\right)}-\gamma_{1}(\pi_{i_{h}\;j_{h}}^{\left(t\right)}-{\bar{\pi }}^{\left(t\right)})+\frac{\gamma_{2}}{H^{\left(t\right)}+K^{\left(t\right)}}{\tilde{\pi }}^{\left(t\right)},\;h=1,2,\ldots ,H^{\left(t\right)}, \end{array}$$

indicating change due to the population density contributed to the dispersion and population density gained as a consequence of dispersion.

The state for non-contributing cells is more complicated to determine since we allow for some of the dispersed population to fail to infect a new host and for the population density in a non-contributing cell that does not receive dispersed population to become zero. We model the potential failure to successfully disperse to a new host using a *dispersion survival probability* with two components. We use a parameter ϕ to determine a ‘baseline’ probability for successful dispersion, and we add a term controlled by a parameter ρ to reflect the assumption that dispersion into cells with non-zero density is more likely to be successful. Recall the MPB depend on overwhelming tree defences for successful invasion. The dispersion survival probability is defined as


(2.3) $$\begin{array}{lcr} & p_{S,ij}^{\left(t\right)}=\phi +\rho \;\arctan(\pi_{ij}^{\left(t\right)}), & \end{array}$$

where $\phi +\frac{\pi }{4}\rho \leq 1$. The shape of the arctangent function means that dispersion is significantly more likely when the receiving cell has a high population density as compared to a low density. This is motivated by the threshold condition in the dispersal–aggregation model in [24]. Then, we define a Bernoulli random variable $B_{S,ij}^{\left(t\right)}$ that determines whether or not dispersion is successful for each non-contributing cell and simultaneously whether the population in the cell becomes zero:


$$B_{S,ij}^{\left(t\right)}=\left\{ \begin{array}{ll} 1, & \text{with probability }p_{S,ij}^{\left(t\right)}\text{,}\\ 0, & \text{with probability }1-p_{S,ij}^{\left(t\right)}\text{.} \end{array}\right.$$

During a simulation, we draw a sample for $B_{S,{\hat{i}}_{k}\;{\hat{j}}_{k}}^{\left(t\right)}$, and only the non-contributing cells with $B_{S,{\hat{i}}_{k}\;{\hat{j}}_{k}}^{\left(t\right)}=1$ successfully receive dispersed density. If dispersion into the cell is not successful, then the population density is zeroed. The state for non-contributing cells becomes


$$\pi_{{\hat{i}}_{k}\;{\hat{j}}_{k}}^{\left(t+1\right)}=\left\{ \begin{array}{ll} \pi_{{\hat{i}}_{k}\;{\hat{j}}_{k}}^{\left(t\right)}+\frac{\gamma_{2}}{H^{\left(t\right)}+K^{\left(t\right)}}{\tilde{\pi }}^{\left(t\right)}, & B_{S,{\hat{i}}_{k}\;{\hat{j}}_{k}}^{\left(t\right)}=1,\\ 0, & B_{S,{\hat{i}}_{k}\;{\hat{j}}_{k}}^{\left(t\right)}=0, \end{array}\right.\;k=1,2,\ldots ,K^{\left(t\right)}.$$

We can generalize the model [29] by allowing the parameters to vary in time and location, specifying a proportional decrease in the cell density rather than zeroing it when dispersion is unsuccessful and by introducing a new ‘shape’ parameter ξ inside the arctangent function:


$$\begin{array}{c} p_{S,ij}^{\left(t\right)}=\phi_{ij}^{\left(t\right)}+\rho_{ij}^{\left(t\right)}\arctan(\xi_{ij}^{\left(t\right)}\pi_{ij}^{\left(t\right)}). \end{array}$$

The parameter ξ can be used to emphasize or de-emphasize the effect of high population density.

#### 2.1.7. Boundary conditions

In the SCA, dispersed populations may move in any direction. Hence, we have to adapt the rules governing dispersion for cells located on the boundary of the domain (figure 2). We impose ‘absorbing’ boundary conditions that effectively allow population to disperse ‘through’ the domain boundary to move outside the domain. (‘Reflecting’ boundary conditions do not allow dispersion to pass the domain boundary.) For each *boundary cell*, or cell with one or more sides on the boundary of the domain, we define *auxiliary cells* adjacent to the boundary cell as needed to create a full Moore neighbourhood for the cell. We alter the update process in a straightforward way (details in [29]) so that the auxiliary cells can receive dispersed density from affected boundary cells, but cannot disperse density to other cells. We note that boundary cells tend to have a lower density than the cells in the interior.

#### 2.1.8. An example of a model update

If we write the state $\left\{\pi_{ij}^{\left(t\right)}\right\}$ as a vector, then after the Bernoulli variables $\left\{B_{I,ij}^{\left(t\right)}\right\}$ and $\left\{B_{S,ij}^{\left(t\right)}\right\}$ have been sampled, we can write the transition as a matrix times the current state. The associated *transition matrix* depends on the current state and time, so the update rule is nonlinear.

**Figure 4.**
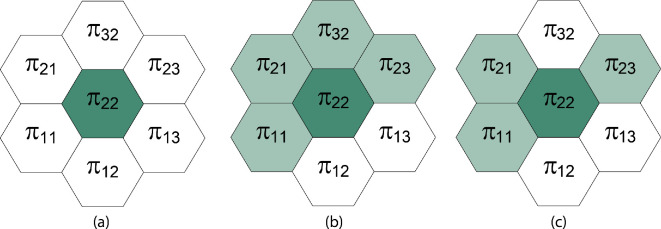
(a) The interaction neighbourhood for the single cell (2, 2). (b) The cells that are included in dispersion after sampling the Bernoulli random variables associated with inclusion in dispersion, showing the affected cell and four non-contributing cells. (c) The cells that successfully receive dispersed density after sampling the Bernoulli random variables associated with successful dispersion.

We provide an example in the case that there is a single affected cell (2, 2). At a given time t, the interaction neighbourhood is the affected cell and its six neighbouring cells (figure 4). We sample the Bernoulli random variables associated with each cell in the interaction neighbourhood with probabilities $\left\{p_{I,ij}^{\left(t\right)}\right\}$.

We assume there are four non-contributing cells included in the dispersion. So the number of contributing cells is H=1, and the number of non-contributing cells is K=4. It follows that


$$\begin{array}{c} \bar{\pi }=\frac{1}{H+K}(\pi_{11}+\pi_{21}+\pi_{22}+\pi_{23}+\pi_{32})=\frac{\pi_{22}}{5}. \end{array}$$

The total amount of population to be redistributed is $\tilde{\pi }=\frac{4\gamma_{1}\pi_{22}}{5}$.

For the contributing cell (2, 2), the density becomes $\pi_{22}-\frac{4\gamma_{1}\pi_{22}}{5}+\frac{4\gamma_{1}\gamma_{2}\pi_{22}}{25}$. For the non-contributing cells, we sample Bernoulli random variable probabilities $p_{S,ij}^{\left(t\right)}=\phi +\rho \;\arctan(\frac{4\gamma_{1}\gamma_{2}\pi_{22}}{25})$ to determine which cells successfully receive dispersed population. Assuming the Bernoulli random variable is 0 for cell (3, 2), its density becomes zero. The cell densities for the other three non-contributing cells are set to $\frac{4\gamma_{1}\gamma_{2}\pi_{22}}{25}$.

We may write this update as a matrix:


$$\begin{array}{c} \begin{array}{r} \left[\begin{array}{c} \pi_{11}^{\left(t+1\right)}\\ \pi_{12}^{\left(t+1\right)}\\ \pi_{13}^{\left(t+1\right)}\\ \pi_{21}^{\left(t+1\right)}\\ \pi_{22}^{\left(t+1\right)}\\ \pi_{23}^{\left(t+1\right)}\\ \pi_{32}^{\left(t+1\right)} \end{array}\right]=\left[\begin{array}{ccccccc} 1 & 0 & 0 & 0 & \frac{4\gamma_{1}\gamma_{2}}{25} & 0 & 0\\ 0 & 1 & 0 & 0 & 0 & 0 & 0\\ 0 & 0 & 1 & 0 & 0 & 0 & 0\\ 0 & 0 & 0 & 1 & \frac{4\gamma_{1}\gamma_{2}}{25} & 0 & 0\\ 0 & 0 & 0 & 0 & 1-\frac{4\gamma_{1}}{5}+\frac{4\gamma_{1}\gamma_{2}}{25} & 0 & 0\\ 0 & 0 & 0 & 0 & \frac{4\gamma_{1}\gamma_{2}}{25} & 1 & 0\\ 0 & 0 & 0 & 0 & 0 & 0 & 0 \end{array}\right]\left[\begin{array}{c} \pi_{11}^{\left(t\right)}\\ \pi_{12}^{\left(t\right)}\\ \pi_{13}^{\left(t\right)}\\ \pi_{21}^{\left(t\right)}\\ \pi_{22}^{\left(t\right)}\\ \pi_{23}^{\left(t\right)}\\ \pi_{32}^{\left(t\right)} \end{array}\right]. \end{array} \end{array}$$

For each t, the state $\{\pi_{ij}^{\left(t\right)}{\}}_{(i,j)\in Z^{2}}$ is a point in a probability space $\left(Q,B_{Q},P_{Q}^{\left(t\right)}\right)$*,* where $B_{Q}$ is the Borel sigma algebra and $P_{Q}^{\left(t\right)}$ is a probability distribution. The explicit update of the state $\{\pi_{ij}^{\left(t\right)}{\}}_{(i,j)\in Z^{2}}$ yields an implicit update of the probability distribution $P_{Q}^{\left(t\right)}$ of the state as well. The implicit update depends on the time, the current state, $\left\{p_{I,ij}^{\left(t\right)}\right\}$*,* $\left\{p_{S,ij}^{\left(t\right)}\right\}$ and the stochastic properties of the microscale information.

We also note that dispersion within distinct interaction neighbourhoods occurs independently. If we write the update rule as a matrix–vector product, the update matrix is sparse and the indexing can be organized so the matrix has a diagonal block structure. We exploit this by a parallel implementation in which the dispersion process is performed for all interaction neighbourhoods simultaneously.

### 2.2. The microscale model

The SCA multiscale framework can accommodate any stochastic microscale model for population dynamics that satisfies three assumptions. First, we assume the microscale model evolves on a fast time scale compared to the macroscale time step. We define a *microscale time step* Δτ=1/N, where N is a large positive integer representing the number of microscale time steps for each macroscale time step. Then, $\tau^{f}=N\times \Delta \tau =1$ is the *final time* for the microscale process over a single macroscale time step. Second, we assume there that the same population dynamics model is used for all cells. Third, we assume that the microscale population dynamics model admits a *carrying capacity* C, or maximum population, on each cell $\kappa_{ij}\in Z^{2}$. In a generalization of the model, N, M and C, as well as any parameters in the population dynamics model, can vary from cell to cell.

For application to MBP dispersion, we use the well-known stochastic birth–death process to describe microscale population dynamics. We stress that any model satisfying the requirements above could be used. A birth–death process is a continuous-time Markov chain that counts the number of individuals in population over time. Let $X_{\tau }$ denote the population size at time τ, which can take values in the finite state space {0,1,2,…,C}*,* where C is the maximum population size (capacity). The rate of births and deaths in a population of size n is determined by instantaneous birth rate $b_{n}$ and instantaneous death rate $d_{n}$, respectively.

Given n individuals at time τ, the probability of a birth in the interval (τ,τ+Δτ)*,* where τ is small, is


$$\begin{array}{c} Pr(X_{\tau +\Delta \tau }=n+1|X_{\tau }=n)=b_{n}\Delta \tau +o(\Delta \tau ), \end{array}$$

where o(Δτ) are terms of smaller order than Δτ. The probability of a death in (τ,τ+Δτ) is


$$\begin{array}{c} Pr(X_{\tau +\Delta \tau }=n-1|X_{\tau }=n)=d_{n}\Delta \tau +o(\Delta \tau ). \end{array}$$

Since Δτ is small, the transition probabilities are


$$Pr(X_{\tau +1}=j|X_{\tau }=i)=\left\{ \begin{array}{ll} b_{i}, & \text{if }j=i+1,\\ d_{i}, & \text{if }j=i-1,\\ 1-b_{i}-d_{i}, & \text{if }j=i,\\ 0, & \text{otherwise.} \end{array}\right.$$

We explore the effect of varying the birth and death rates in §3.4.

### 2.3. Coupling the microscale and macroscale models

In general, multiscale models must address two challenges. First, it is necessary to ‘translate’ information relevant to processes at one scale into a form that is relevant to processes at the other scale. For example in the MPB model, the microscale (tree scale) model describes population dynamics, while the macroscale SCA model describes changes to population density. Second, the dynamics at each scale have to be synchronized with the dynamics at the other scale. For example, in the MPB model, the population dynamics of MPB on trees occurs much faster than the effective time needed to see significant changes at the macroscale due to dispersion. These issues can have a significant impact on the computational time needed for model simulation. Generally, the coupling of microscale and macroscale models in a multiscale model is a cottage industry in many applications.

#### 2.3.1. Simulation of the microscale model

Because the microscale model is stochastic, we use the expectation of the microscale population to couple to the macroscale model. In practice, we estimate the expectation with an average over a finite number of simulations. We let M denote the number of independent simulations of the microscale model on any cell $\kappa_{ij}$. We denote the collection of simulations of the microscale model on cell $\kappa_{ij}$ by $\{X_{ij,\ell }^{\left(\tau \right)}{\}}_{\ell =1,\tau =0}^{M,N}$*,* where $X_{ij,\ell }^{\left(\tau \right)}$ takes values in state space {0,1,2,⋯,C}. We assume that the $X_{ij,\ell }^{\left(\tau \right)}$*,* ℓ=1,⋯,M, evolve independently, so the microscale simulations can be carried out in parallel.

#### 2.3.2. Translation between scales and synchronization

**Figure 5.**
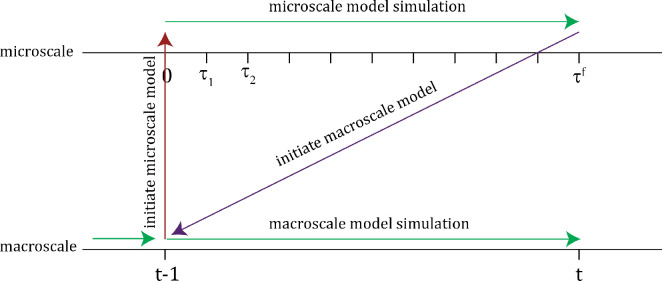
Illustration of the microscale–macroscale coupling algorithm.

Typical approaches to simulate multiscale models use the concept of ‘discrete synchronization’. Based on the assumption that the microscale model evolves on a faster time scale, at a macroscale time step t−1 (a *synchronization time*), the macroscale state is converted to an initial condition for the microscale model. The microscale model is simulated to the next synchronization time holding any macroscale quantities constant. Upon completion of the microscale simulation, the microscale state is converted to compute an updated macroscale state at time t−1. The macroscale model is then simulated over the next macroscale time step. We illustrate this in figure 5. There are a number of other possible algorithms, e.g. updating the macroscale model halfway between synchronization times and iterating the communication between microscale and macroscale simulations several times before proceeding to the next synchronization time [30]. Tests of several approaches did not yield significantly different results for the SCA applied to MPB dispersion.

To be precise for our approach, at macroscale time t−1, the macroscale density $\pi_{ij}^{\left(t-1\right)}$ on cell $\kappa_{ij}$ is used to initiate M independent realizations of the model simulation by setting $X_{ij,\ell }^{\left(0\right)}=\pi_{ij}^{\left(t-1\right)}\times C$*,* ℓ=1,⋯,M. Then, we evolve M simulations of the microscale model independently to reach the final values $X_{ij,\ell }^{\left(\tau_{ij}^{f}\right)}$*,* ℓ=1,⋯,M. We use the expected values of the final values to update the macroscale density state $\pi_{ij}^{\left(t-1\right)}$. Namely, we set


$$\pi_{ij}^{\left(t-1\right)}=\frac{1}{M}\sum_{\ell =1}^{M}\frac{X_{ij,\ell }^{\left(1\right)}}{C}.$$

We then simulate the SCA from t−1 to t.

### 2.4. Implementation and simulation

We use the R programming language (v. 4.1.1) to build a simulator that takes an initial cell configuration and a set of parameter values for the SCA transition rules and outputs the cell configuration evolution at a sequence of times (see [29,53] for details and algorithms).

The simulations were executed on the Compute Canada’s high-performance computation system. The maximum number of cells, and so the effective physical size of the region, on which the multiscale model for dispersion can be used depends primarily on computational capacity. The density of trees and the variability in conditions for the trees are also factors determining the maximum size of the region that can be studied with a given computational capacity. The model can be applied with a single tree in each cell, in which case it is modelling dispersion between individual trees. However, each cell can include a number of trees provided that it is reasonable to simulate the birth–death process on the trees in the cell with the same parameters and dispersion among the trees in the cell takes place in an approximately uniform fashion. When this is valid, it means the physical size of the region on which dispersion is modelled is larger.

The domains in the simulations consist of 30×30 and 50×50 non-overlapping cells in most cases. Since each realization is independent of the other and no effect is needed to communicate between the realizations, we use parallel computing to carry out the simulations simultaneously. When we investigate the evolution patterns of two interaction neighbourhoods, we use apply functions in R to simulate the dispersion process for two groups at the same time.

Experimentally, we found that M=20 provided reasonable estimates for the expected population from the stochastic birth–death process in each cell. Increasing M increases the simulation time. We generally choose C=100 and N=100, which allows the birth–death process to approach steady state. Small changes to M and N do not yield significantly different behaviour in the multiscale dispersion model.

## 3. Investigations of model behaviour

In this section, we conduct a numerical investigation into the properties and behaviour of the multiscale SCA model. The focus of the investigation is understanding how model behaviour depends on various parameters and inputs. Since the parameters of the SCA model are determined by Bayesian calibration from observational data, we consider the parameters as random in some studies and investigate the effect of changing their distribution. More extensive investigations are presented in [29].

We summarize the parameters for the multiscale model for dispersion of MPB in table 1. Unless noted, we assume the same parameter values in all cells in a given simulation. We state the parameter values used for the simulation study in table 2.

**Table 1.** Parameters in the multiscale model for MBP dispersion.

parameter	information
$\gamma_{1}$	fraction of population that tries to disperse
$\gamma_{2}$	the rate of successful dispersion to a potential host
ϕ,ρ	determines the dispersion survival probability for infestation of a new host
b	parameter determining the birth rate in the birth–death process
d	parameter determining the death rate in the birth–death process
C	the carrying capacity for the birth–death process
M	number of simulations of the microscale model in each cell
N	number of microscale time steps per macroscale time step

**Table 2.** Parameter settings for simulation studies.

	SCA model				birth–death process				
varying	$\gamma_{1}$	$\gamma_{2}$	ϕ	ρ	b	d	N	C	M
birth–death process	0.8	0.8	1/3	1/3	—	—	100	100	20
$\gamma_{1}$	—	0.8	1/3	1/3	1	0.2	100	100	20
$\gamma_{2}$	0.8	—	1/3	1/3	1	0.2	100	100	20
survival probability	0.8	0.8	—	—	1	0.2	100	100	20

### 3.1. Statistics for quantifying properties of dispersion patterns

In order to quantify the evolution of dispersion patterns, we compute statistics that quantify key properties of the patterns. The goal is to determine statistics that can be computed for data from experimental observation and provide the basis for calibration of model parameters. At the macroscale, key data include images and measurements of dispersion patterns at different times.

First, we use the change in the area of affected cells to measure the ‘rate of dispersion’. For each interaction neighbourhood, we construct a circle that has the same area as the affected cells in an interaction neighbourhood centred at its centre of mass. The centre of mass is calculated using the position of the cells in the interaction neighbourhood weighted by their density. To define the rate of dispersion, we compute the change in the radius of the circles for an interaction neighbourhood as it evolves from one time to the next.

**Figure 6.**
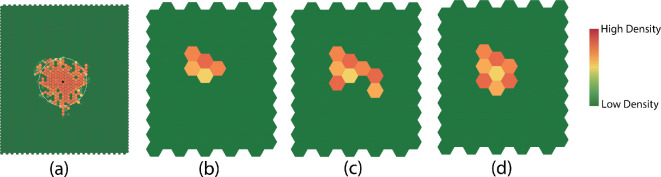
(a) The circle and centre of mass of an interaction neighbourhood. (b) An interaction neighbourhood with five affected cells. The total number of boundary edges is 16 and the complexity of the pattern is 16/5=3.2. (c,d) Interaction neighbourhoods with the same number of cells but different complexities. The complexity for (c) is 3.5, and the complexity for (d) is 2.6.


Second, we define two statistics that quantify the ‘complexity’ of a dispersion pattern in an interaction neighbourhood. Both statistics are computed using the boundary of the interaction neighbourhood. In the first approach, we define the measure of complexity as the number of boundary edges divided by the number of affected cells in the interaction neighbourhood. The complexity is larger if there is complex geometry in the boundary (figure 6). The second approach to computing complexity is used when the interaction neighbourhood becomes extremely large. For each affected cell in the interaction neighbourhood, we count the number of its adjacent affected neighbours $n_{a}$, where $a=\left\{1,\ldots ,N_{a}\right\}$*,* and $N_{a}$ is the total number of affected cells in the pattern. The formula for computing complexity is as follows:

$$\text{complexity}=\left(6N_{a}-\sum_{a=1}^{N_{a}}n_{a}\right)/N_{a}.$$

There are identical numbers of affected cells in the interaction neighbourhoods shown in figure 6c,d, but they have different shapes. The total number of boundary edges is 28 for pattern (c) and 21 for pattern (d). Thus, the complexity for two patterns is 3.5 and 2.6, respectively. In general, the complexity is smaller for patterns with a ‘smoother’ boundary.

To compute these statistics, we choose 1000 random samples for the parameters and then simulate the multiscale model 50 times for each sample. We conduct the experiments with the minimum grid size capable of producing perceptible patterns that are sensitive to different parameter settings, which is typically 30 × 30 grids or larger. In cases where experiments require more than 30 time steps, we use a 50 × 50 grid that allows for evolution lasting up to 120 time steps before significant boundary effects occur.

### 3.2. General observations

**Figure 7.**
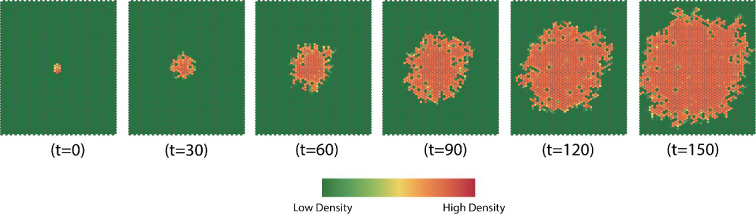
Typical evolution of dispersion. Generally, under homogeneous conditions, dispersion patterns form roughly ellipsoidal shapes after moderate time has passed. The boundaries of the affected region are complex due to the stochastic nature of dispersion. Similarly, there are interior cells that have low density. The rate of expansion of the affected area and the complexity of the boundary are determined by the parameters in the model.

**Figure 8.**
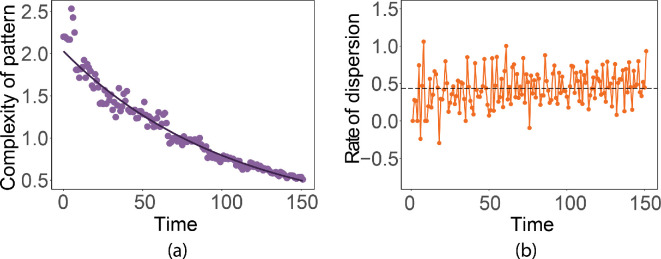
(a) Complexity versus time. We fit a curve to the values $\left(\ln(\text{complexity})=0.71-0.01\times \text{time},R^{2}=0.97,F(1,150)=5211,p<0.001\right)$. (b) Dispersion rate. The black dashed line is the average rate over all simulations.

We begin with observations about general behaviour. In figure 7, we show the evolution of a typical simulation at six times. Figure 8 displays the evolution of the complexity and rate.

The progress of dispersion in the simulation is typical of behaviour for parameter values that yield expanding dispersion. The expanding regional is roughly ellipsoidal in shape, but the boundary of the affected region forms a heterogeneous pattern reminiscent of figure 1b. Additionally, there are cells in the interior that have low population density because the cell is not included in the dispersion process and/or the cell population died out as a consequence of the failure of dispersed population to find a host in the cell. At t=150, the affected area is just reaching the boundaries, so future evolution would be affected by the boundary conditions.

**Figure 9.**
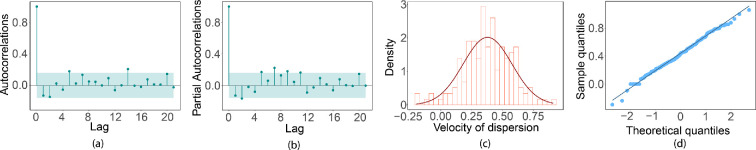
Dispersion rate data. (a) ACF. (b) PACF. (c) eCDF plot. (d) Normal *QQ* plot.

In general, the complexity decreases exponentially with time, as the cells near the boundary represent a decreasing fraction of the total affected cells (figure 8). The dispersion rate oscillates around a straight line. According to the autocorrelation plot (ACF) and partial autocorrelation plot (PACF) for the dispersion rate data in figure 9, there is no apparent structure in the data, while the ACF and PACF plots suggest the data constitute white noise. A Box–Ljung test $\left(\chi^{2}=29.30,df=20,p=0.08\right)$ suggests that the dispersion rate data resembles white noise and the empirical cumulative distribution function (eCDF) plot, the normal *QQ* plot and the Kolmogorov–Smirnov test for normality (D=0.11,p=0.33) all support the conclusion that the data resemble Gaussian white noise.

In subsequent simulations, we carried out these analysis steps and reported on the conclusions. The detailed results can be found in [29].

Due to the stochastic nature of the model, the evolution patterns do not repeat even with identical initial patterns and parameter settings. Multiple simulations with the same parameter values and initial conditions yield very similar complexity and dispersion statistics [29].

### 3.3. Investigation of the effect of varying parameters and conditions

**Figure 10.**
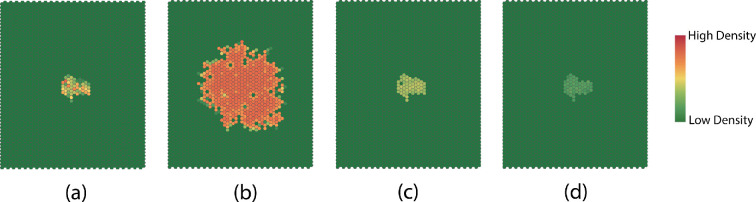
Typical examples of late time dispersion patterns for different regimes of parameters: (a) initial pattern, (b) unbounded growth, (c) steady state and (d) fade away.

We focus the study on quantities that have a significant effect on the dynamics of the SCA. Extensive simulation studies [29] suggest that the behaviour approaches the following three limiting scenarios for affected regions: (i) growth without limit; (ii) convergence to patterns that vary with roughly fixed size; and (iii) fade away completely. These are illustrated in figure 10.

#### 3.3.1. Varying distributions of the parameters

**Figure 11.**
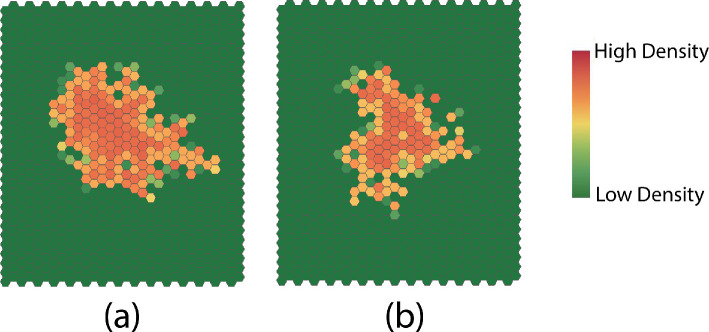
Typical late time dispersion patterns for (a) experiment 1 and (b) experiment 2. The parameter distribution in experiment 2 leads to significantly more complex patterns in general.

Considering parameters as random, we set up two experiments with different prior distributions on parameters in the model to investigate the effect on dispersion. In the first experiment, we assign a normal distribution N(0.8,0.4/6) for both $\gamma_{1}$ and the dispersion survival rate $\gamma_{2}$ while holding the other parameters fixed. In the second experiment, we assign a normal prior N(0.8,0.4/6) for $\gamma_{1}$, and a normalized beta prior beta(2, 4) for ϕ in the dispersion survival probability with ϕ∈[1/6,5/6] holding the other parameters fixed (we set ρ=1/12 to reduce the effect of cell density). We show typical patterns of simulations at later times from the two experiments in figure 11.

The results for the first experiment suggest that the average number of affected cells, the average density, and the complexity are all normally distributed. In the second experiment, the average number of affected cells and the average density appear to be distributed to a gamma distribution, while the complexity is distributed to a skew generalized error distribution. We also see significant differences in the dispersion patterns for the two experiments. We conclude that varying the distribution of the parameters leads to patterns with different statistical properties. Differences are also evident in the patterns shown in figure 11.

#### 3.3.2. Varying the fraction of population $\gamma_{1}$ that is dispersed

**Figure 12.**
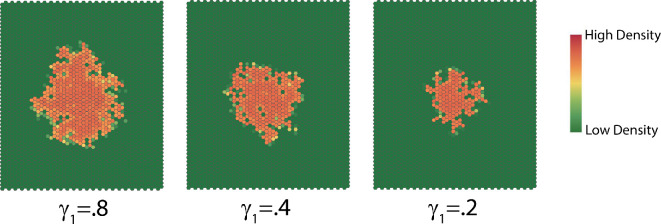
Typical dispersion patterns at time t=50 generated for various values of $\gamma_{1}$.

**Figure 13.**
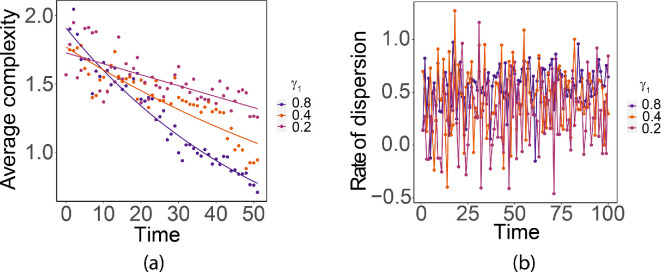
(a) Complexity with $\ln(\text{complexity})=0.42-0.01\times \text{time},R^{2}=0.98,F(1,99)=6697,p<0.001$; $\ln(\text{complexity})=0.53-0.01\times \text{time},R^{2}=0.99,F(1,99)=25180,p<0.001$; and $\text{complexity}=1.69-0.01\times \text{time},R^{2}=0.99,F(1,99)=16720,p<0.001$ for $\gamma_{1}$ equal to 0.8,0.5 and 0.2, respectively. (b) Dispersion rates.

The parameter $\gamma_{1}$ is the fraction of population that is dispersed. As we observe in figure 12, the size of the interaction neighbourhood at a given time increases when $\gamma_{1}$ increases, which is verified by the rate statistics (figure 13). The patterns resulting from different $\gamma_{1}$ are similar. Decreasing $\gamma_{1}$ has a significant effect on the rate at which the complexity decreases.

#### 3.3.3. Varying the dispersion survival rate $\gamma_{2}$

**Figure 14.**
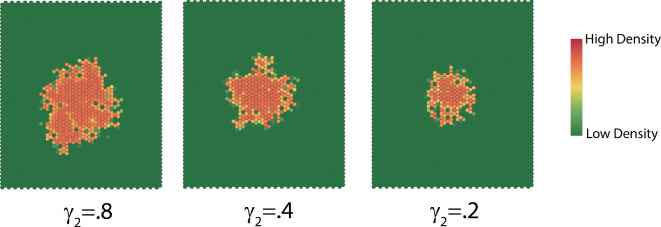
Typical dispersion patterns at t=50 generated with different dispersion survival rates $\gamma_{2}$.

**Figure 15.**
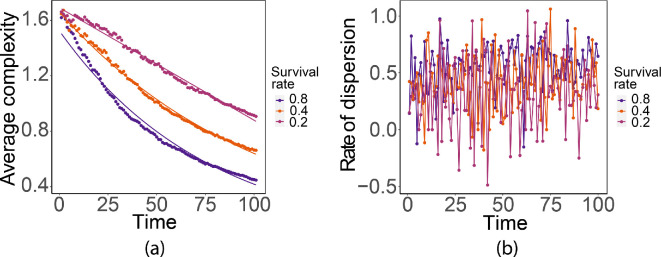
(a) Complexity with ln⁡(complexity)=$\;0.42-0.01\times \text{time},R^{2}=$0.98,F(1,99)=6697,p<0.001;ln⁡(complexity)=$\;0.5-0.01\times \text{time},R^{2}=0.99,F(1,99)=$22210,p<0.001,ln⁡(complexity)=$\;0.55-0.01\times \text{time},R^{2}=$0.99,F(1,99)=11380,p<0.001 for survival rate $\gamma_{2}$ equal to 0.8,0.4 and 0.2, respectively. (b) Dispersion rates.

The dispersion survival rate $\gamma_{2}$ determines the fraction of the population that successfully disperses to new hosts. In figure 14, we see that a larger dispersion survival rate leads to a faster increase in the size of the interaction neighbourhood. The complexity decreases exponentially for the three dispersion survival rates (figure 15), with faster decrease for dispersion larger survival rate, and the dispersion rate resembles Gaussian white noise.

#### 3.3.4. Varying the dispersion survival probability

**Figure 16.**
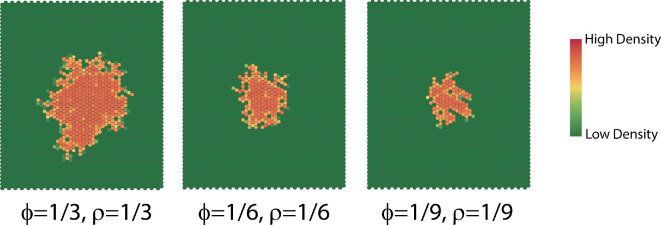
Dispersion patterns at t=50 generated with different survival probabilities.

**Figure 17.**
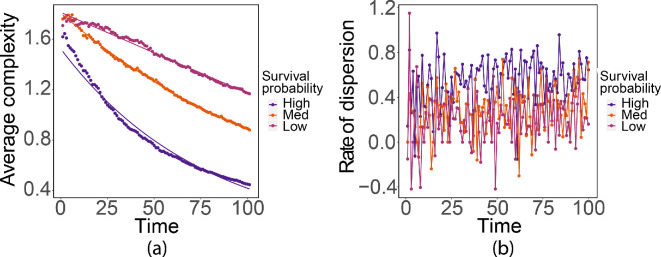
(a) Complexity with ln⁡(complexity)=$\;0.42-0.01\times \text{time},R^{2}=$0.98,F(1,99)=6697,p<0.001;ln⁡(complexity)=$\;0.6-0.01\times \text{time},R^{2}=$0.99,F(1,99)=49100,p<0.001;complexity=$\;1.8-0.006\times \text{time},R^{2}=0.98,F(1,99)=7267,p<0.001$ for large, medium and small dispersion survival probabilities, respectively. (b) Dispersion rates.

The dispersion survival probability (2.3) depends on the population density that is received by the non-contributing cells. The interaction neighbourhood grows faster, and there are fewer vacant cells inside the interaction neighbourhood when the dispersion survival probability is large (figure 16). This is because a large dispersion survival probability means that the population is more likely to successfully disperse into the new hosts. The complexity decreases linearly when the dispersion survival probability is at a very low level (figure 17). The evidence suggests that the dispersion rate resembles Gaussian white noise.

#### 3.3.5. Inhomogeneous physical conditions

**Figure 18.**
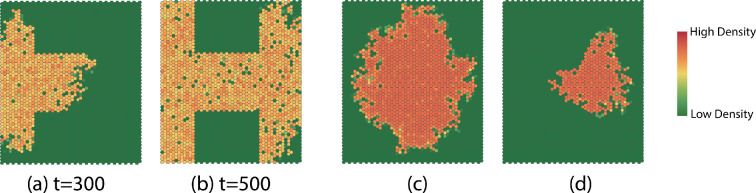
Dispersion patterns for inhomogeneous conditions. (a,b) The case when there is a narrow region where dispersion can take place. (c,d) Dispersion patterns at t=120 including the effects of wind. (c) Dispersion is favoured in all directions equally. (d) Dispersion to the east is favoured.

Physical environments may have inhomogeneous conditions that affect dispersion. For example, deforested areas such as prairie, grassland, rocky hills, large lakes or human constructions and weather conditions such as wind affect dispersion of MPB. We model a situation in which the region under consideration is divided into two parts connected by a ‘narrow bridge’ in which dispersion can take place (figure 18). The population disperses through the indicated region, but it takes much longer to spread than in previous examples.

To model the effect of wind, we vary the probability of inclusion in dispersion using the general form (2.2) according to relative position. For example, if the wind is blowing from west to east, we specify probabilities to increase the chance of including high-density cells on the west and low-density cells on the East while lowering the probability of including low-density cells on the west and high-density cells on the east. We see in figure 18 that the population tends to disperse to the east. Dispersion also occurs more slowly than in previous examples.

### 3.4. The effect of the microscale population dynamics model on dispersion

The multiscale model for dispersion is constructed specifically to provide a way to link macroscale dispersion to a microscale population model. Including the microscale model greatly increases the complexity and cost of simulations. In this section, we provide evidence that the microscale model can have a very significant impact on dispersion at the macroscale.

#### 3.4.1. Varying parameters in the birth–death process

**Figure 19.**
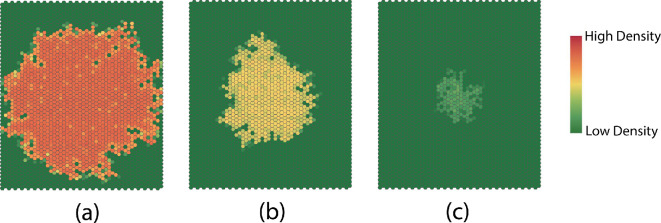
Dispersion patterns at time t=100 generated with different death rates in the birth–death process: (a) d=0.2; (b) d=0.5; (c) d=0.8.

We assume a linear death rate $d_{n}=dn$ and a logistic birth rate $b_{n}=nb(1-n/C)$, where C is the capacity. Fixing all the other parameters in the multiscale model and setting b=1, we investigate the effect of varying the death rate d. As we observe in figure 19, the microscale death rate affects the complexity and the dispersion rate.

**Figure 20.**
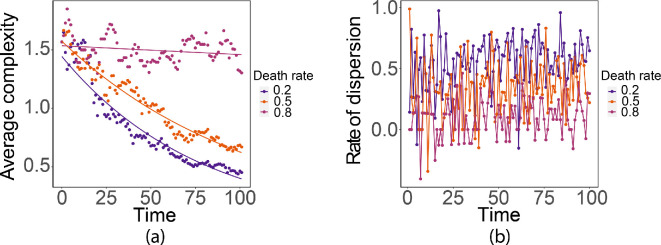
(a) Complexity with $\text{complexity}=1.67-0.002\times \text{time},R^{2}=0.88,F(1,99)=697,p<0.001$*,* ln⁡(complexity)=$\;0.42-0.01\times \text{time},R^{2}=$0.98,F(1,99)=6697,p<0.001;ln⁡(complexity)=$\;0.49-0.01\times \text{time},R^{2}=$0.99,F(1,99)=10980,p<0.001 for death rates 0.8,0.5 and 0.2. (b) Dispersion rates.

The complexity decreases linearly when the death rate is large. When the death rate is small, the interaction neighbourhood expands at a much higher rate (figure 20).

**Figure 21.**
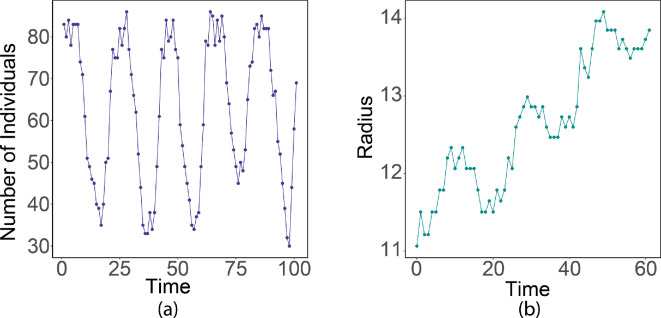
(a) Number of individuals generated from the birth–death process over time. (b) Radius of the circle that equals the total area of all affected cells in the interaction neighbourhood.

Recalling that the MPB is univoltine with a life cycle that is correlated annually with the temperature, we let the birth–death process have annual cyclic behaviour by fixing the death rate d=0.2 and setting a periodic birth rate $b_{n}=nb(1-n/C)+nb(1-n/C)\;\sin(2t\pi /T)$ with b=1 and a T=20 (corresponding to dividing a year into 20 macroscale time steps; figure 21). Consequently, the macroscale cell density and dispersion patterns exhibit cyclic behaviour. For example, the radius of the circle used to compute the rate of dispersion in an interaction neighbourhood exhibits a cyclic behaviour (figure 21).

#### 3.4.2. The long time endemic–epidemic cycle

One important and fascinating aspect of MPB dynamics is that infestations may remain dormant in an endemic phase for many years before an epidemic phase in which the population ‘explodes’ or increases at a dramatically faster rate. Such epidemics seem to be correlated with a change in environmental conditions, such as a period of hotter years and drought [18–22]. These kinds of environmental conditions have a dramatic effect on the tree-scale population dynamics of MPB. In this section, we investigate whether or not the microscale model dynamics can cause dispersion behaviour akin to the endemic–epidemic cycle. This is important because the environmental conditions are most directly reflected in the parameters of the population dynamics model.

**Figure 22.**
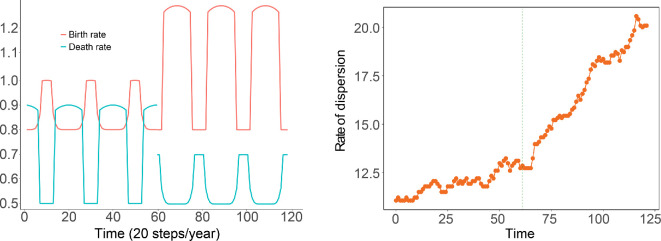
(a) Periodic birth rate and death rate for the birth–death process. (b) Rates of dispersion.

We set up periodic birth rates and death rates in the microscale birth–death process for two regimes (figure 22). In endemic years, we assume the birth rate of MPB is large only during a very short time during the summer, while the death rate is higher than the birth rate during the rest of the year because of cold weather. In epidemic years, we assume the birth rate is higher than the death rate throughout the year because MPB is more likely to survive when the temperature is at a higher level.

We compute simulations that span dormant to epidemic years, modelling a relatively cool period followed by a series of hotter years. We simulate the first 60 time steps using the birth–death process parameters for dormant years and the subsequent 60 time steps using birth–death process for the epidemic years (figure 22).

**Figure 23.**
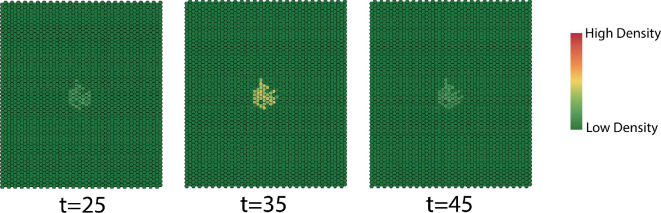
Dispersion patterns at indicated times during endemic years.

**Figure 24.**
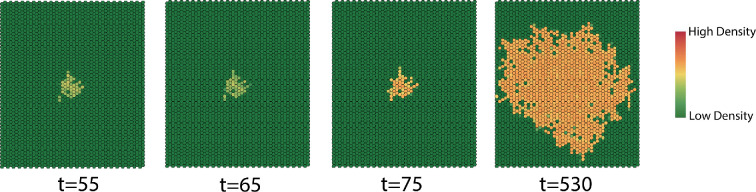
Dispersion patterns at indicated times during epidemic years.

In figure 22, we see that the rate of dispersion varies over the first 60 time steps. The size of the interaction neighbourhood grows when the birth rate is higher than the death rate and decreases when the birth rate is smaller than the death rate. The dispersion patterns in figure 23 provide the same conclusion. During the epidemic years phase, the interaction neighbourhood grows throughout the year since the birth rate is much higher than the death rate, which reflects the MPB dispersion when the epidemics start. The infestation eventually disperses to the entire domain. The evolution patterns in figure 24 provide the same conclusion.

## 4. Parameter calibration

Given a multiscale model of complex phenomena and probability distributions for the parameters in the model, the behaviour of the phenomena can be predicted using Monte Carlo sampling. Namely, we can draw sample values of the parameters from their distributions, simulate the model for those values and evaluate a quantity of interest, e.g. statistic, from the model solutions to get a collection of (simulation) data. The data can be used to compute an empirical probability measure (the ‘push-forward’ measure) for the quantity of interest, from which we can draw probabilistic inferences, such as the probability of an event.

This standard framework for prediction requires distributions on model parameters. That can lead to challenging problems. In some cases, parameters may be measured by direct observation or measurement. But often, the only knowledge about parameters that is available comes from observation of system behaviour. This leads to the *calibration problem* of inferring information about parameters in a model from observational data on behaviour of the model [54]. That is why an important property of any model is the degree to which the parameters in the model can be ‘calibrated’ or determined from observed data on the behaviour being modelled.

The *calibration problem* is a form of inverse problem for the map that implicitly relates the model parameters to the model output. Generally, such maps have the property that multiple parameter values yield the same output data, so assumptions are required in order that the calibration problem admits a well-defined solution. In addition, the multiscale model for dispersion of MPB poses three challenges for calibration.

*The coupling of microscale and macroscale models*: In general, parameter calibration for multiscale models is an open research question [55–57]. The issue is that the upscaling and downscaling of information required to couple models at different scales typically involves a significant loss of information, e.g. by averaging. For example, it is often difficult to calibrate parameters in a microscale model using data from macroscale behaviour.

We approach this challenge by assuming that the parameters in the tree-scale population dynamics model for MPB are calibrated directly from field observations using the population dynamics model. We only discuss calibration of parameters in the macroscale SCA model. The fact that there is a great deal more observational data for the tree-scale dynamics of MPB than for dispersion supports this approach.

*The stochastic nature of the model*: A complication with calibration for the SCA is the stochastic nature of the output, which means that repeated simulations with the same parameters may yield different results. We borrow an idea from the analysis of time series. In time series, the first step is to use data to estimate the deterministic ‘trend’ in the data, then to model the residual data obtained by subtracting the trend as stochastic noise. We calibrate the model results for the expectation of the stochastic SCA model outputs. Specifically, we calibrate with respect to the output of the SCA model obtained by averaging over a large number of simulations.

*The available data for quantifying dispersion*: A challenge in modelling dispersion of MPB are that there is limited data quantifying the spread of MPB infestation [23,34,58–65]. We devise a method to calibrate from graphical images of the spread of dispersion by adapting the classic Bayesian model for noisy observations.

### 4.1. General formulation and solution of the calibration problem

To formulate the probability model for the parameters, we use λ as a generic name for parameters in the SCA model taking values in the parameter domain Λ. We use Q(λ) to denote the expected value of an ‘observed quantity’ or statistic measured from the solution of the SCA at parameter value λ that corresponds to experimental data. So Q is the map from the parameter domain Λ to the *observable space* D={Q(λ):λ∈Λ}. In this framework, experimental data correspond to random sampling from the probability model for the parameters. We assume that there is an (unobserved) *data-generating distribution* ${\tilde{P}}_{\Lambda }$ on Λ. Each random sample, $\lambda ∼{\tilde{P}}_{\Lambda }$ yields an observed datum q=Q(λ). The calibration problem is thus: given a set of observed data $\{q_{i}=Q(\lambda_{i}){\}}_{i=1}^{n}$ corresponding to a set of sample parameter values $\{\lambda_{i}{\}}_{i=1}^{n}$, determine a *posterior probability distribution* $P_{\Lambda }$ such that $\{\lambda_{i}{\}}_{i=1}^{n}$ is a random sample from $P_{\Lambda }$. Because many combinations of parameters in the SCA model lead to identical dispersion patterns, there are multiple solutions to the calibration problem, and we do not expect $P_{\Lambda }$ to equal ${\tilde{P}}_{\Lambda }$.

A challenge for calibration of dispersion for MPB using real data is that we have perhaps only one datum or at most a small collection of data for a specific region. In this case, we adopt the classic statistics approach of calibrating data obtained by adding stochastic observation noise to the given dispersion pattern. This corresponds to a situation in which there is a ‘true’ set of parameter values, but the value is uncertain. This approach is partially motivated by the observation that a data-generating distribution that is concentrated near a point produces output data that are similarly distributed near a single output value if Q is well-behaved. The investigation in §3.3 .1 suggests this holds for the SCA. We model the output as $\tilde{q}=Q(\tilde{\lambda })+\epsilon$, where ϵ is a random noise with distribution N(0,Σ) with fixed covariance Σ, and λ is the true value of the parameter for the observed behaviour. Then, given a collection of observed noisy model output values $\{{\hat{q}}_{i}{\}}_{i=1}^{n}$, we compute a posterior SIP solution $P_{\Lambda }$ on a chosen Λ that induces the random observations. We can estimate the true value from $P_{\Lambda }$, e.g. by computing the mean.

There are a variety of approaches for calibration of model parameters from observed data, and the literature for calibration in both application domains and statistics is very large. We cite just a few recent review references [38,66–68] as background. Adopting a Bayesian approach is fairly common [69–71]. In this paper, we adopt the *stochastic inverse problem* (SIP) approach [72–76] to Bayesian calibration. The review paper [54] discusses the differences between the SIP and other approaches to Bayesian calibration. Upon choice of a *Bayesian prior*, representing foreknowledge about parameter values, the *Bayesian posterior* solution of the SIP, representing updated information gained from experimental observation, is unique and depends continuously on parameters and data. We use the non-informative ‘uniform ansatz’ prior to determining the solution [72–76]. The resulting solution uses the least prior knowledge (maximum entropy) among all possible solutions. The solution of the SIP is approximated by a form of importance sampling, implemented in the public domain Python code BET [77], which is used for the computations below. Details of the solution of the calibration problem can be found in [29,53].

### 4.2. Selection of parameters for calibration

An important step in defining a calibration problem in practice is identification of those parameters that can be calibrated from the model and the available data and those parameters that can be determined directly or through another experiment. If a parameter can be measured directly, it should be removed from the calibration problem to reduce the dimension of the parameters involved in calibration. If the quantity of interest (statistic) computed from the model output is insensitive to a particular parameter, that parameter may be very difficult to calibrate regardless of approach. The investigations in §3.3 show that $\gamma_{2}$ and ϕ have very similar effect on the statistics of dispersion. This suggests that it will be difficult to distinguish between ϕ and $\gamma_{2}$ in calibration. For this reason, we fix $\gamma_{2}$. We can equally fix ϕ or set $\phi =\gamma_{2}$ and perform calibration. The second observation is that the choice of ρ has much less effect on the statistics of dispersion than ϕ, which means it is likely to be difficult to calibrate ρ if both ϕ and ρ are included in the calibration. We fix ρ.

### 4.3. Two calibration problems

We conduct two investigations of the calibration problem for the multiscale model of dispersion. In the first investigation, we use ‘simulated’ data, which means that we have as much data as desired for the calibration. In the second investigation, we use real data in the form of photographic evidence of dispersion. We have limited data in this case, so we adopt the classic Bayesian approach of considering noisy observations of the real data.

We list the parameter values for the calibration experiments in table 3.

**Table 3.** Parameter settings for calibration.

SCA model				birth–death process				
$\gamma_{1}$	$\gamma_{2}$	ϕ	ρ	b	d	N	C	M
—	0.8	—	1/3	1	0.2	100	100	20

We use 30 macroscale time steps for the calibration.

#### 4.3.1. Calibration using simulated data

In the first investigation, we use simulated data. We specify distributions for the parameters to be calibrated, simulate the model by random sampling the distributions to generate data, ‘forget’ the initial distributions for the parameters and then use the Bayesian calibration to compute a posterior distribution for the parameters. One goal of this experiment is to compare calibration results for various statistics computed from the data.

We assume a normal data-generating distribution ${\tilde{P}}_{\Lambda }=N((0.7,\frac{1}{3}{)}^{⊤},0.05^{2})$ that is centred at $\gamma_{1}=0.7$ and $\phi =\frac{1}{3}$. We generate 1000 samples of $\gamma_{1}$ and ϕ and compute 50 solutions of the SCA for each sample, then compute the average statistics from the resulting dispersion patterns. This yields 1000 observed data $\{q_{i}{\}}_{i=1}^{1000}$ for the SIP. We then ‘forget’ ${\tilde{P}}_{\Lambda }$ and compute a Bayesian posterior SIP solution $P_{\Lambda }$ using the non-informative ‘uniform ansatz’ prior. Note that we do not expect $P_{\Lambda }$ to equal ${\tilde{P}}_{\Lambda }$.

We consider four statistics computed from dispersion patterns for the inversion:

(1) Average number of affected cells.
(2) Average cell density.
(3) Complexity of dispersion pattern.
(4) Derivative of a fitted curve for complexity.

We emphasize that these statistics are chosen because they can be computed for graphical images of real MPB infestations. We wish to determine if these statistics are reasonable choices for the purpose of Bayesian calibration.

**Figure 25.**
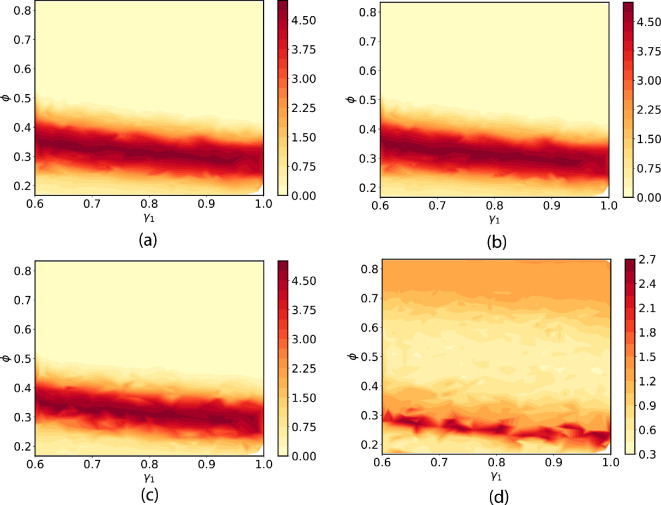
Heatmaps for the density of the Bayesian posterior solution computed from four quantitative statistics. (a) Average number of affected cells. (b) Average cell density. (c) Average complexity of dispersion pattern. (d) Derivative of fitted curve of complexity.

Heatmap plots of the density for the Bayesian posterior solution $P_{\Lambda }$ are shown figure 25. The solutions corresponding to statistics (1), (2) and (3) are similar while the solution for statistic (4) is quite different. In all cases, the plots show a density that has the form of a ‘ridge’ that is roughly parallel to the $\gamma_{1}$ axis. This indicates that the distribution of $\gamma_{1}$ is nearly uniform over the domain. Reflecting the uniform ansatz Bayesian prior, these solutions indicate that the values of $\gamma_{1}$ in Λ are all equally likely to be consistent with the data, and we cannot resolve the value $\gamma_{1}$ beyond the resolution provided by the range of values in Λ using any of the four statistics.

In contrast, the ϕ cross-section of the SIP posterior distribution presents a much different conclusion. For statistics (1), (2) and (3), the cross-section is highly localized into a relatively small region around 0.3 within the bounds on ϕ given by Λ. The corresponding density is highly ‘peaked’ within this small region. This indicates that it is possible to calibrate ϕ with relatively high precision using the first three statistics. There is more complex structure in the density for statistics (4). Namely, the cross-section for ϕ has a bi-modal shape with peaks in the density of the posterior around 0.26 and 0.74. There are two intervals of ϕ values that are consistent with the observed data on the statistics to high probability. This means that statistics (4) provides less information about ϕ than the other statistics, and we omit it when calibrating with real data.

The solutions of the SIP for the first three statistics indicate that it is possible to calibrate ϕ with much higher precision that $\gamma_{1}$. This is not surprising given that the numerical investigations suggest that dispersion patterns are relatively sensitive to the choice of ϕ.

#### 4.3.2. Calibration using a photograph of damage due to mountain pine beetle infestation

**Figure 26.**
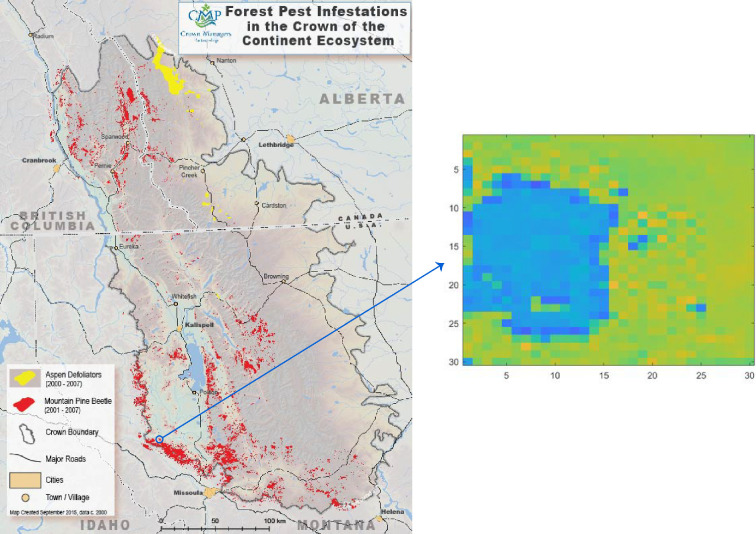
Left: an aerial survey map for MPB infestation from 2001 to 2007 in the Crown of the Continent Ecosystem [60]. Right: scaled bitmap corresponding to the indicated region.

For calibration using real data, we use an aerial survey that quantifies the severity of an MPB infestation in a region used to monitor the long-term ecological health of the Crown of the Continent Ecosystem (figure 26). The map indicates the dead pine trees in red from MPB attacks during the period 2001–2007. At the time of the image, the area of dead pine trees occupied about 4.4% of the entire Crown of the Continent Ecosystem region [60].

In order to perform calibration, we convert this survey map into a numerical field. The original aerial survey map represents 2500 × 3800 km region rendered as a JPG image with 1291×1455 pixels. We use Photoshop to crop the image to a region of 32 × 32 pixels, representing a region of 58 × 58 km (figure 26). Given this is several years into an infestation, we chose a cropped region in which the dispersion pattern is roughly ellipsoidal. We avoid a pattern that is sparsely heterogeneous since this is characteristic of early stages of dispersion in that region and we do not know the initial conditions. We indicate the chosen region with a blue circle in figure 26.

In order to obtain a population density, we convert the colour jpeg image into grey scale with 256 shades and store the result in a matrix corresponding to a domain with a 32×32 grid. Dividing the entries by 256 yields a density for each cell of the grid. To focus on the dead trees in the image, we zero cell densities below the specified threshold of 0.8, which effectively removed colours other than red.

To obtain the ‘noisy’ data for calibration, we add iid mean zero normally distributed random values to each non-zero value in the matrix. Specifically, $N(0,0.2^{2})$ noise is added to the density in the cells that are non-zero, and $N(0,0.6^{2})$ noise is added to the density cells that are adjacent to at least one non-zero density cell. Finally, if a cell has density that is above 1, then we set it to 1; and if a cell has density that is below 0, we set it to 0.

**Figure 27.**
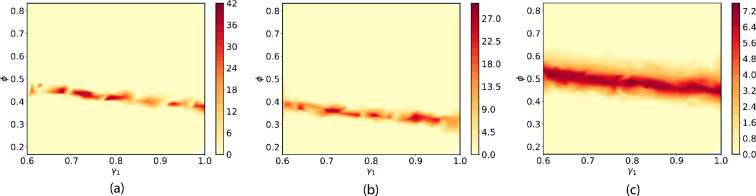
Bayesian posterior density contour plot for $\gamma_{1}$ and ϕ generated from three quantitative measurements. (a) Average number of affected cells. (b) Average cell density. (c) Average complexity of dispersion pattern.

We use the three statistics (1), (2) and (3) for calibration. We plot heatmaps of the density of the Bayesian posterior SIP solution corresponding to the uniform ansatz prior in figure 27. The solutions for statistics (1) and (2) are quite similar, while the solution for statistics (3) is different from that of the other two. In all cases, we see the nearly uniform distribution for $\gamma_{1}$ in the chosen range that we found when solving the SIP with simulated data. This indicates that $\gamma_{1}$ cannot be resolved beyond the chosen range. Similarly, the ϕ cross-section for all three solutions is limited to a relatively small interval within the range specified by Λ and is highly peaked. There is ≈10% difference in the ranges for ϕ yielded by the solutions corresponding to statistics (1) and (2). The range for ϕ for statistic (3) is shifted considerably and is much broader.

## 5. Conclusion

We construct a multiscale SCA model for dispersion of a population driven by a microscale stochastic model for population dynamics. The macroscale dispersion model and microscale model population dynamics model are coupled using a sequential approach that assumes that dispersion occurs on a slower time scale than population dynamics. A primary motivation is to construct a model for dispersion that uses simple rules and a few parameters to generate complex behaviour.

We apply the model to MPB infestation in western North American forests. The numerical investigation of the model behaviour shows that the multiscale dispersion model can yield a wide range of behaviours and can produce patterns that are qualitatively similar to those of MPB infestations. This includes demonstrating that changes in the microscale model parameters due to changing environmental conditions can drive the characteristic dormant-epidemic dynamics of MPB infestations. We investigate the sensitivity of dispersion patterns, quantified by statistics related to the complexity and rate of dispersion, to changes in parameters and physical conditions. We also solve the Bayesian calibration problem to determine key parameters in the dispersion model from experimental data.

There are several interesting directions for future research.

The SCA model is formulated in a general fashion so that it can be readily adapted to other situations involving dispersion of populations. Likewise, the multiscale framework can easily accommodate different microscale models for population dynamics, and it would be interesting to investigate the effect of using different models on dispersion dynamics. Also worth investigating is the incorporation of various techniques for suppressing MBP infestations into the model.

The numerical investigation suggests that the SCA has some persistent qualities, e.g. stability with respect to various perturbations, and it would be interesting to conduct a theoretical analysis. However, this is challenging because the multiscale SCA is highly nonlinear.

**Figure 28.**
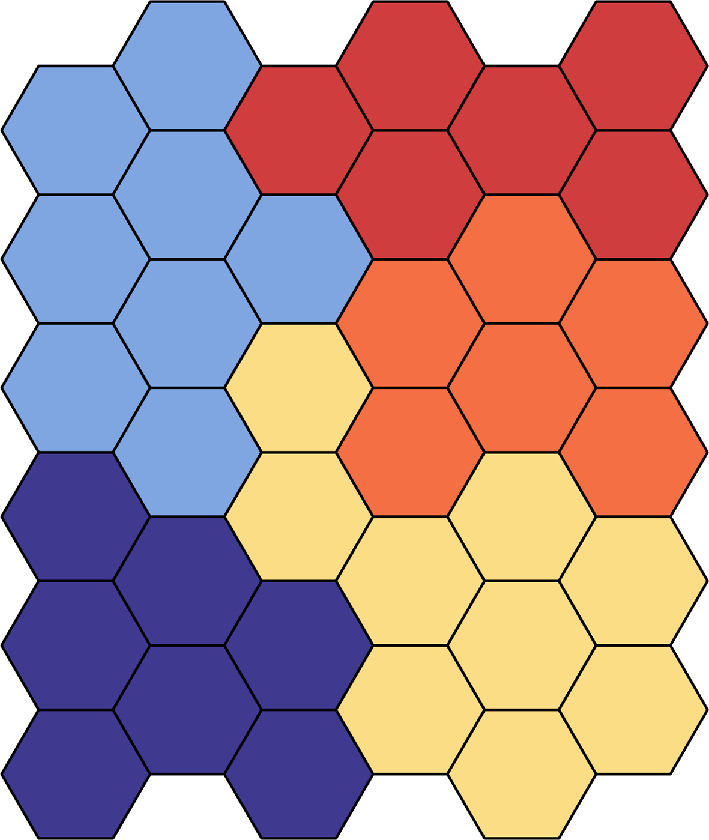
A plot of cascaded SCA model. The hexagons represent cells in the ‘child’ unit, and the hexagons of the same colour form the interaction neighbourhoods of the ‘child’ unit. The interaction neighbourhoods of the ‘child’ unit form the cells of the ‘parent’ unit.

One possible subject of further research is to include more than two scales in the SCA model by constructing a ‘cascaded’ SCA model. For example, in figure 28, we assume individual behaviour occurs within each cell, and the cells of the same colour belong to the same interaction neighbourhood with highly similar physical properties. We call cells of the same colour the ‘child’ unit and the entire region the ‘parent’ unit. We implement the SCA model within each ‘child’ unit, in which the ‘child’ unit for colouring areas does not intersect. We treat each ‘child’ unit as cells in the ‘parent’ unit and use the output of the ‘child’ unit as the input for the SCA model on the ‘parent’ unit. This cascaded SCA model can be performed efficiently in parallel. The interest in this approach is that it can be applied to a much larger region.

## Data Availability

Data and relevant code for this research work are stored in GitHub (https://github.com/Irene-liu-724/MPB-Infestation) and have been archived within the Zenodo repository (https://zenodo.org/account/settings/github/repository/Irene-liu-724/MPB-Infestation). The data (an image) used in the paper is available at: https://www.sciencebase.gov/catalog/item/560312d0e4b03bc34f5449aa.
